# An exploration of Northern Ireland general practice pharmacists’ views on their role in general practice: a cross-sectional survey

**DOI:** 10.1186/s12875-024-02457-7

**Published:** 2024-06-06

**Authors:** Abrar H. F. Hassan, Heather E. Barry, Carmel M. Hughes

**Affiliations:** 1 Primary Care Research Group, School of Pharmacy, Medical Biology Centre, Queen’s University Belfast, 97 Lisburn Road, Belfast, BT9 7BL Northern Ireland, UK; 2 Department of Clinical Pharmacy, College of Pharmacy, Jazan University, Jazan, Saudi Arabia

**Keywords:** Primary care, General practice, General practice pharmacists, Cross-sectional, Northern Ireland

## Abstract

**Background:**

There is limited research examining the views of general practice pharmacists (GPPs) on their role and their impact in general practice. The aim of this study was to explore GPPs’ views regarding this role and its potential impact within general practice in Northern Ireland (NI).

**Methods:**

A paper-based self-administered questionnaire was mailed to 319 general practices in NI in 2022, directed to the GPP who spent most time at the practice. A variety of closed and open questions were included in six sections. Responses to closed questions were analysed descriptively whilst open question responses were analysed using content analysis. To ascertain associations between variables (e.g. GPP prescribing status, working arrangements and aspects of collaboration with GPPs), Fisher’s exact test was employed with an a *priori* significance level of *p* < 0.05.

**Results:**

155 responses were received equating to a response rate of 48.5%. Most participants (72.3%) were female, independent prescribers (71%), and 64.5% were currently using their independent prescriber qualification. Services that were provided by most GPPs were medication reconciliation (99.4%) and medication reviews (97.4%). The most common method of communication between GPPs and general practitioners (GPs) was face-to-face (89.0%). Telephone was the most common method of communication between GPPs, community pharmacists (97.4%) and patients (98.7%). Most GPPs (> 80%) showed positive attitudes towards collaboration with GPs and those who worked in multiple practices were more likely to agree with the Attitudes Towards Collaboration Instrument for pharmacists (ATCI-P) statements compared to those who worked in a single practice (*p* < 0.05). Less than 40% (36.8%) of GPPs agreed that patients were aware of the role they provided. The majority of GPPs (80.6%) expressed positive views on their impact on primary care. Analysis of the free-text comments revealed the need for more GPP patient-facing activities, GPP-specific training, and promotion of the GPP role.

**Conclusion:**

The findings indicated that GPPs had largely positive views about their role and their impact on primary care. The results may be helpful for practices and service commissioners. Further research is necessary to explore the perspectives of patients regarding the role of the GPP and to enhance patients’ awareness of the GPP.

**Supplementary Information:**

The online version contains supplementary material available at 10.1186/s12875-024-02457-7.

## Background

Primary care serves as the initial point of access for healthcare for most patients and encompasses a range of essential services, including general practice, dentistry, optometry, and community pharmacy services [1]. The staff in general practice are varied, such as general practitioners (GPs), practice nurses, receptionists, administrative staff, health visitors, midwives, allied health professionals, and social workers [2]. The current and expanding need for patient care among individuals with multimorbidity (the coexistence of two or more chronic conditions) and polypharmacy (the simultaneous use of five or more medications) has resulted in a two- to three-fold increase in primary care consultation rates in the United Kingdom (UK) when compared to European countries [3–5]. According to a recent study conducted in the UK, there has been a notable increase in the number of GP consultations and consultations with other healthcare professionals (HCPs) in general practice in the time frame spanning from April 2000 to March 2019 [6]. Specifically, the median number of all GP consultations has risen from 13 to 21 per patient per annum, while all consultations with other practice staff have gone from a median of 27 to 60 per patient per annum [6]. This has been further aggravated by a decrease in the number of GPs because of factors associated with recruitment and retirement [7]. To alleviate this burden, various pilot initiatives were introduced in 2015 in England and Northern Ireland (NI) with the aim of tackling the fundamental concerns regarding staffing and workload in primary care [7, 8]. These pilot initiatives were designed to help general practices to incorporate supplementary healthcare professionals [9]. One such measure involved the incorporation of pharmacists within general practice, commonly referred to as general practice pharmacists (GPPs) [9]. The pilot initiatives conducted in England and NI not only aimed to reduce the workload in general practice and increase the workforce, but also aimed to promote the management of chronic health conditions and foster collaboration among primary care team members [7, 8]. The pilot initiative of the National Health Service in England (NHS England) was implemented with the objective of recruiting 470 GPPs in over 700 general practices [7]. This initiative was backed by funding of £31 million [7]. Subsequently, an extra allocation of £100 million was provided to facilitate the expansion of integration efforts, encompassing an additional 1,500 GPPs within general practices [7, 10]. These GPPs are expected to assume both clinical and non-clinical responsibilities, while actively participating as members of the primary care multidisciplinary team [7, 10]. According to NHS England, the pilot initiative allocated funding to support the employment of pharmacists for a duration of three years [7, 11]. Specifically, the funding covered 60% of the costs incurred in the first year (2015), 40% of the costs in the second year (2016), and 20% of the costs in the third year (2017) [7, 11]. Thereafter, it was requested that general practices assume responsibility for the remaining expenses associated with the employment of pharmacists in general practice [7, 11]. Moreover, the incorporation of pharmacists into general practice is projected to experience further growth via Primary Care Networks (PCNs) by the year 2024 [12]. The Department of Health in NI allocated a financial investment of £17 m to support the integration of pharmacists within general practices [13]. It was projected that by the year 2020/2021, there would be an estimated total of 300 whole time equivalent GPPs in employment [14]. At the end of the pilot initial phase in NI for the financial year 2020/2021, an additional £18 million was allocated to sustain financing of 303 whole-time equivalent GPPs for the year 2021/2022 [13]. Given the developing nature of the role of GPPs, it is imperative to conduct additional research to have a comprehensive understanding of the perspectives of GPPs on their integration into general practice in NI. A previous investigation into the perspectives and attitudes of GPs in NI regarding GPPs revealed that most GP participants held favourable views and attitudes towards GPPs [15]. This study highlighted the necessity of investigating the perspectives of GPPs regarding their role to validate these results [15]. Therefore, this current study, with a focus on GPPs aimed to investigate: (1) GPP attitudes towards collaboration with GPs; (2) GPPs’ roles in general practice and how these roles were agreed on; (3) GPPs’ views about their role and its impact upon primary care; and (4) GPPs’ views on communication with patients and patients’ awareness of their role.

## Methods

### Study design, sample, and setting

A cross-sectional study was conducted with GPPs in NI general practice. At the time of this study, there were 319 active general practices and 2,715 registered pharmacists in NI [13, 16]. 12% (*n* = 325) of these pharmacists were GPPs according to the information provided by the Department of Health and the former Health and Social Care Board [13, 16], indicating that every general practice has at least one GPP [15, 16]. The study was reported using the Consensus-Based Checklist for Reporting of Survey Studies (CROSS) (Additional file 1) [17].

### Questionnaire construction and content

The development of the questionnaire was informed by a comprehensive examination of the existing literature pertaining to the perspectives of GPPs, their understanding of their professional roles within the context of general practice, and the nature of their collaborative relationships with GPs [10, 18–22]. The content of the questionnaire was also informed by prior research undertaken by members of the research team. This earlier research involved the completion of self-administered questionnaires by community pharmacists and GPs in NI, with the aim of assessing their perspectives on GPPs [15, 23]. The questionnaire comprised of six distinct sections (see Additional file 2), denoted as A, B, C, D, E, and F. The first section of the questionnaire (A) gathered non-identifiable demographic information from participants (such as gender and age), thereby providing descriptive characteristics of the sample. Section B gathered data on the activities undertaken by GPPs and the process of selecting these activities. The third section (C) examined the communication patterns among GPPs, GPs, community pharmacists, and other staff members within the general practice. This involved assessing the frequency of meetings between these groups and the methods of communication used. Additionally, this section explored the underlying reasons for communication. In section D, the Attitudes Towards Collaboration Instrument for pharmacists (ATCI-P) was utilised to assess the attitudes of GPPs towards collaborating with GPs [24]. This instrument had been developed based on a review of literature on interprofessional collaboration and interviews conducted with pharmacists and GPs [24]. The ATCI-P consists of 15 items that require respondents to express their level of agreement or disagreement using a five-point Likert scale [24]. Thirteen statements were included in this questionnaire, with two statements being excluded as they were deemed irrelevant to the research aim. In the fifth section (E), a five-point Likert scale was employed to assess the perspectives of GPPs regarding patients’ encounters with GPPs. This encompassed aspects such as the understanding of the GPP’s role, communication methods, and the level of trust established between GPPs and patients. The last section (F) examined the extent to which GPPs agreed or disagreed on the impact of their role on patients and GPs in primary care, using a five-point Likert scale. This included the effects of the GPP role on patient outcomes, general practice workload, and the wastage of medicines. At the end of the questionnaire, space was provided where participants could record any other comments (free-text responses). The questionnaire did not undergo any reliability or validity assessment since the pilot testing of the questionnaire had a limited number of participants (see below). Nevertheless, the pilot phase, as described below, aided in addressing specific problems related to face validity.

### Questionnaire piloting

The questionnaire underwent piloting with four pharmacists, two of whom had prior experience working as GPPs. Participants were asked to provide general feedback and suggestions regarding the layout and content of the questionnaire, and duration for completion. According to the pilot responses, minor modifications were applied to the questions and the cover letter of the questionnaire to specify the rationale for the research and provide clearer instructions for questionnaire completion. The expected duration for completing the questionnaire was 15 min. The pilot responses were not included in the final sample and analysis.

### Questionnaire administration

A paper-based self-administered questionnaire was distributed to all general practices (*n* = 319) in NI on two occasions (June and July 2022). This method of data collection had been used previously with GPs in a related study and had generated a good response rate (61.7%; [15]). A cover letter accompanying the questionnaire gave a brief description of the research’s context and aim. It stipulated that it was intended for completion by the GPP who spent most of their time working in that practice and that the GPP should respond to the questionnaire only once. If the GPP allocated their time equally among many general practices, they were requested to fill out the questionnaire once, and their responses pertained to their experience at one specific general practice. Furthermore, the cover letter explicitly stated that participants in this study who completed the questionnaire should not include any identifiable information, such as the names of their GP colleagues, when providing their responses to the given questions. The cover letter informed participants of the confidentiality and anonymity of their collected data. GPPs who submitted completed questionnaires were considered to have provided implicit consent. The consent process was approved by the Queen’s University Belfast Faculty of Medicine, Health, and Life Sciences Research Ethics Committee-see later. The cover letter stipulated a designated deadline by which completed questionnaires were to be returned, i.e. two weeks after initial posting, using a pre-paid envelope. Following a further two weeks (i.e. four weeks after the original posting), a reminder letter including an additional copy of the questionnaire, cover letter and pre-paid return envelope were posted to all general practices in NI. Recipients were directed to complete the questionnaire if they had not already done so.

### Statistical analysis

Questionnaire data were coded and inputted into a Microsoft Excel spreadsheet. Double-checking of 10% of data entry was undertaken by a member of the research team (HEB) to verify the accuracy and integrity of the data, hence minimising the presence of any potential errors. The data were imported to SPSS version 28.0 for analysis [25]. Descriptive analyses, such as frequencies and proportions, were employed to provide a comprehensive description of the participants and their corresponding responses. Associations were examined between questionnaire variables such as the prescribing status of the GPP (i.e. whether the GP possessed an independent prescriber qualification and whether they were currently utilising this qualification), and their working arrangements (specifically, the number of practices the GP was employed in and the number of sessions worked per week) and other variables including aspects of collaboration using Fisher’s exact test, with significance set *a priori* at *p* < 0.05. Missing responses were not considered in the final analysis.

### Analysis of free text responses

Free-text responses were compiled in Microsoft Word and analysed using a broad approach by the research team member (AHFH) [26, 27]. The responses were read repeatedly to gain an understanding of meaning. Thereafter, the responses were broadly categorised to allow presentation of the main findings. No inter-rater reliability determined as only one member of the research team undertook this process, although another member of the research team checked the categories for face validity (CMH).

## Results

Following the first mailing, 103 responses (32.3%) were received. After the second mailing, 52 responses (16.2%) were received. In total, 155 responses were received from both mailings of the questionnaire, equating to a response rate of 48.5% (155/319).

### Demographic data

A summary of the demographics and working environment details of the GPP participants is provided in Table 1. From the total of 155 participants, 112 identified as female (72.3%) and 42 identified as male (27.1%). Most participants were categorised into two age groups: 30–39 years (60.6%, *n* = 94) and 40–49 years (25.8%, *n* = 40). Two-thirds of GPPs surveyed (65.8%, *n* = 102) had a postgraduate qualification and 71.0% (*n* = 110) reported they were independent prescribers. A significant proportion of GPPs (64.5%, *n* = 100), were found to be actively utilising their independent prescriber qualification. A total of 80 GPPs (51.6%), reported working in multiple general practices. Similarly, 79 participants (50.9%) indicated that they dedicated four to six work sessions (refers to the designated periods of time that GPPs spend working in a general practice, with one session equating to approximately four hours) each week to the general practice where they mostly worked. Over half of the participants (55.5%, *n* = 86) had between one to four years of experience working in general practice as GPP. Additionally, 57.4% of participants (*n* = 89) had also worked for one to four years in the general practice where they spent most of their time. Having an independent prescriber qualification or using the independent prescriber qualification was not associated with the number of general practices in which GPPs worked (*p* = 0.590 and *p* = 0.143, respectively). Likewise, there was no association between the number of sessions that GPPs worked in general practice and their possession or use of their independent prescriber qualification (*p* = 0.435 and *p* = 0.106, respectively). It was reported by almost 60% of GPPs (59.4%, *n* = 92) that they had a consulting room available for them to use either always or very often; 23.2% (*n* = 36) reported it was sometimes available, and 17.4% (*n* = 27) reported it was rarely or never available. The location of the general practice [as denoted by Trust area (of which there are five) within NI] where GPPs spent most of their time was: Belfast Trust 19.4% (*n* = 30), South-Eastern Trust 16.8% (*n* = 26), Northern and Southern Trusts 24.5% (*n* = 38) each, and Western Trust 14.2% (*n* = 22). Just under half (48.4%, *n* = 75) of the practices where GPPs worked were located in urban areas, 32.3% (*n* = 50) were in suburban areas, and 18.7% (*n* = 29) were in rural areas. The final question in the demographics section enquired about other pharmacy sectors in which GPPs had previously worked: 72.2% (*n* = 148) had prior experience working in a community pharmacy, 13.2% (*n* = 27) had worked in a hospital pharmacy, while 2.9% (*n* = 6) had previous employment in the pharmaceutical industry.

**Table 1:** GPP participant demographic and practice profile

Demographics	*N*	%
Gender		
Female Male Prefer not to say Other	112 42 1 0	72.3 27.1 0.6 0
Age (years)		
< 30 30–39 40–49 50–59 ≥ 60	10 94 40 11 0	6.5 60.6 25.8 7.1 0
GPP postgraduate qualification		
Yes No Missing	102 50 3	65.8 32.3 1.9
GPP Independent Prescriber qualification		
Yes No	110 45	71.0 29.0
Use of Independent Prescriber qualification		
Yes No Missing Not applicable	100 8 2 45	64.5 5.2 1.3 29.0
Number of general practices at which GPP worked		
1 2 3 > 3	61 80 12 2	39.4 51.6 7.7 1.3
Number of sessions worked per week where GPP spent most of time		
1–3 4–6 7–9 10 Missing	10 79 38 19 9	6.5 50.9 24.5 12.3 5.8
Years of working as GPP		
< 1 1–4 5–9 ≥ 10	11 86 46 12	7.1 55.5 29.7 7.7
Years of working as GPP at general practice where GPP spent most of time		
< 1 1–4 5–9 ≥ 10	24 89 36 6	15.5 57.4 23.2 3.9
Availability of a consulting room for GPP where GPP spent most of time		
Always Very often Sometimes Rarely Never	61 31 36 25 2	39.4 20.0 23.2 16.1 1.3
Trust area of general practice where GPP spent most of time		
Belfast Northern South-Eastern Southern Western Missing	30 38 26 38 22 1	19.4 24.5 16.8 24.5 14.2 0.6
Location of general practice where GPP spent most of time		
Rural Suburban Urban Missing	29 50 75 1	18.7 32.3 48.4 0.6
*Other sectors of pharmacy where GPP had worked		
Community pharmacy Hospital pharmacy Academia Pharmaceutical industry Other (specified by GPPs): - Commissioners of services (e.g. CCG-Clinical Commissioning Group). - Clinical research/clinical trials. - Prison pharmacy. - Professional body. - Research ethics/ regulations. - Education and training.	148 27 11 6 13	72.2 13.2 5.4 2.9 6.3

GPP: general-practice-pharmacist * Respondents could select more than one sector of pharmacy where they had worked in the past

### Activities of general practice pharmacists

Participants were asked to describe the activities that GPPs undertook (summarised in Table 2) and the process by which these activities were allocated to them. Medication reconciliation (99.4%, *n* = 154), medication reviews (97.4%, *n* = 151), counselling patients to help them manage their medications (96.8%, *n* = 150), and reauthorising repeat prescribing (95.5%, *n* = 148) were the activities undertaken by most GPPs. The least provided activities by GPPs were educational group sessions to healthcare providers (17.4%, *n* = 27), research (8.4%, *n* = 13), outreach involvement (5.8%, *n* = 9), and educational group sessions to patients (2.6%, *n* = 4). The responses regarding how GPP activities were allocated varied greatly, but for most GPPs (90.3%, *n* = 140), their professional activities in general practice were decided upon by mutual agreement between the GPP and the GP. The GPP’s level of confidence (69.7%, *n* = 108), the GPP’s present skills (79.4%, *n* = 123), the GP Federation (60.6%, *n* = 94) (a group of general practices that collaborate to establish an organisational unit and operate within a certain geographic region; the GP federation offer the GPP terms and conditions of employment, as well as occupational maternity pay and sick leave benefits [28]), and the GPP’s prior experience (53.5%, *n* = 83) also influenced the decision on the allocation of professional activities. In addition, GPPs who chose the ‘Other’ option (4.5%, *n* = 7) stated that their professional activities were decided by the Federation and the GPP by mutual agreement, the GPP’s qualification, the general practice manager, or GPs (often without the GPP’s mutual agreement), the GPP’s areas of interest, the needs of the practice, or the work done by the previous pharmacist in the practice.

**Table 2:** Activities undertaken by GPPs in general practice

Activity	*N* (%)
Medication reconciliation.	154 (99.4)
Patient medication queries.	154 (99.4)
Medication reviews.	151 (97.4)
Counselling patients to help them manage their medications.	150 (96.8)
Reauthorising repeat prescribing.	148 (95.5)
Answering medicines information enquiries from health care providers.	147 (94.8)
Educating patients on how to take their medicines.	146 (94.2)
Addressing medicines adherence with patients.	144 (92.9)
Managing other issues that involve medication such as adverse drug reactions and drug-drug interactions.	140 (90.3)
Conducting audits as part of the multidisciplinary team.	138 (89.0)
Administrative duties such as dealing with outpatient clinical letters and hospital discharge letters.	137 (88.4)
Signposting patients to appropriate services and other healthcare professionals (e.g. community pharmacists).	130 (83.9)
Counselling patients in relation to lifestyle interventions.	125 (80.6)
Developing guidelines and/or practice formulary.	109 (70.3)
Running clinics with patients (e.g. asthma, blood pressure, vaccination).	102 (65.8)
Acute prescribing.	94 (60.6)
Student training.	56 (36.1)
Triaging and managing minor ailments.	46 (29.7)
Educational group sessions to healthcare providers.	27 (17.4)
Research.	13 (8.4)
Outreach involvement (e.g. Drug and Therapeutics Committee).	9 (5.8)
Educational group sessions to patients.	4 (2.6)
Other (specified by GPPs): - Improving safety (e.g. amber/high risk drug monitoring). - Improving quality (e.g. working with other HCPs to address issues with medicines). - Improving efficiency and cost effectiveness (e.g. conduct searches through the practice clinical system-EMIS® web and develop recommendations for safe and cost-effective prescribing). - Team-working (e.g. communicate with community pharmacists regarding queries, blister packs/medication changes/ medication shortages).	37 (23.9)

GPPs: general-practice-pharmacists, HCPs: healthcare professionals, EMIS®: Egton Medical Information Systems

### Communication of GPPs within primary care

Regarding the common and preferred method of communication between GPPs and GPs, a higher percentage of GPPs reported that face-to-face was both the most common (89.0%, *n* = 138) and preferred (87.7%, *n* = 136) method of communication as illustrated in Additional file 3. In relation to the frequency of face-to-face meetings between GPPs and GPs, more than half of participants (56.8%, *n* = 88) indicated that they engaged in daily face-to-face communication with GPs (see Additional file 3). Additionally, GPPs provided a list of the common reasons for GPPs to communicate with GPs and for GPs to communicate with GPPs such as medication and prescribing issues (see Additional file 4).

In contrast to the common and preferred method of communication between GPPs and GPs, the predominant form of communication between GPPs and community pharmacists was via telephone, as reported by GPPs. This method of communication was reported as the most common by 97.4% (*n* = 151) of GPPs and as the preferred method by 93.5% (*n* = 145) of GPPs, as depicted in Additional file 3. Regarding the frequency of face-to-face contact between GPPs and community pharmacists, GPPs reported variations in the frequency of meeting face-to-face with community pharmacists as shown in Additional file 3. Only 4.5% (*n* = 7) of respondents reported that they had daily face-to-face contact with community pharmacists and 17.4% (*n* = 27) chose the ‘Other’ option, i.e. never, rarely, and face-to-face contact for local pharmacies only (see Additional file 3). Almost all GPPs (98.7%, *n* = 153) reported common reasons for GPPs to communicate with community pharmacists and common reasons (97.4%, *n* = 151) for the community pharmacists to communicate with GPPs (see Additional file 4).

Additionally, GPPs reported that they had communicated with multiple health and social care professionals both within and outside the general practice. Specifically, 99.4% (n = 154) of GPPs had interactions with the reception staff, 96.8% (n = 150) had interactions with both practice nurses and practice managers, and 27.1% (n = 42) had interactions with pharmacy technicians (see Additional file 3). Furthermore, 37.4% (n = 58) selected the ‘Other’ option and disclosed engaging in communication with nurses (e.g. advanced nurse practitioners and palliative care nurses) and allied health professionals (e.g. dieticians and cognitive behavioural therapists).

### Attitudes towards collaboration with general practitioners

Using the ATCI-P, the vast majority of GPPs (> 80%) had positive attitudes towards collaboration with GPs, with the majority of GPPs agreeing or strongly agreeing with every statement on the ATCI-P. Most participants (98.8%, *n* = 153) agreed or strongly agreed that collaboration between the GPP and the GP improved patient care; the majority of GPPs (98.8%, *n* = 153) agreed or strongly agreed that patients benefitted from the collaboration. Equal numbers of GPPs agreed/strongly agreed that professional communication between the GPP and the GP was open and honest (97.5%, *n* = 151) and the GP believed that the GPP had a role in assuring medication safety (97.5%, *n* = 151). GPP responses to the ATCI-P statements are displayed in Table 3.

**Table 3:** GPPs’ responses to the ATCI-P statements

Statement	Agree/Strongly agree *N* (%)	Neither agree nor disagree *N* (%)	Disagree/Strongly disagree *N* (%)
1. The professional communication between myself and the GP is open and honest.	151 (97.5)	1 (0.6)	3 (1.9)
2. The GP is open to working together with me on patients’ medication management.	149 (96.1)	2 (1.3)	4 (2.6)
3. The GP has time to discuss with me matters relating to patients’ medication regimens.	128 (82.6)	13 (8.4)	14 (9)
4. I meet the professional expectations of the GP.	144 (92.9)	8 (5.2)	3 (1.9)
5. The GP trusts my professional decisions.	145 (93.6)	9 (5.8)	1 (0.6)
6. Discussions with the GP help me provide better patient care.	153 (98.8)	0 (0)	2 (1.2)
7. The GP and I have mutual respect for one another on a professional level.	147 (94.8)	6 (3.9)	2 (1.3)
8. The GP and I share common goals and objectives when caring for the patient.	148 (95.5)	6 (3.9)	1 (0.6)
9. My role and the GP’s role in patient care are clear.	131 (84.5)	15 (9.7)	9 (5.8)
10. The GP has confidence in my expertise.	141 (91.0)	13 (8.4)	1 (0.6)
11. The GP believes that I have a role in assuring medication safety (for example, to identify drug interactions, adverse reactions, contraindications etc.)	151 (97.5)	3 (1.9)	1 (0.6)
12. The GP believes that I have a role in assuring medication effectiveness (for example, to ensure the patient receives the optimal drug at the optimal dose etc.)	150 (96.8)	4 (2.6)	1 (0.6)
13. My working together with the GP benefits the patient.	153 (98.8)	1 (0.6)	1 (0.6)

GP: general practitioner

Those who worked in multiple practices were more likely to express agreement with 11 out of 13 ATCI-P statements in respect of collaboration compared to those who worked in a single practice (Fisher’s exact test *p* < 0.05) as shown in Table 4 and Additional file 5.

**Table 4:** GPPs’ perceptions of patients’ awareness of the GPP role

Statement	Agree/Strongly agree *N* (%)	Neither agree nor disagree *N* (%)	Disagree/ Strongly disagree *N* (%)	Missing *N* (%)
Patients are aware of the role I provide.	57 (36.8)	46 (29.7)	51 (32.9)	1 (0.6)
Patients are aware of the difference between the GPP role and the community pharmacist role.	39 (25.2)	46 (29.7)	69 (44.5)	1 (0.6)
Patients trust my ability to provide high-quality care.	121 (78.1)	28 (18.1)	5 (3.2)	1 (0.6)
My professional activities are valued by patients.	126 (81.4)	23 (14.8)	5 (3.2)	1 (0.6)

GPP: general-practice-pharmacist

### General practice pharmacists’ views on communication with patients and patients’ awareness of their role

In relation to the interaction between GPPs and patients in the context of general practice, it was found that the predominant method used by GPPs for communication with patients was the telephone. This method was reported as the most common by 98.7% of GPPs (*n* = 153), and it was also the preferred method of communication for most participants (92.3%, *n* = 143) (see Additional file 3). GPPs were also asked about the frequency with which they engaged in direct, face-to-face interactions with patients. There was significant variation in responses with 10.3% (*n* = 16) of GPPs reporting daily face-to-face contact with patients as indicated in Additional file 3. Just over 70% of participants (72.3%, *n* = 112) reported the main concerns raised during interactions with patients included medication issues and reviews, prescribing issues, management of long-term conditions and education on medications and health conditions.

This section also examined the perceptions of GPPs on patients’ awareness of their role, as depicted in Table 4. Just over one-third of GPPs (36.8%, *n* = 57) agreed or strongly agreed that patients were aware of the GPP’s role. Furthermore, 25.2% of GPPs (*n* = 39) agreed or strongly agreed that patients were aware of the distinction between the role of a GPP and that of a community pharmacist.

### Views on the impact of general practice pharmacists in primary care

Most participants (97.5%, *n* = 151) expressed agreement or strong agreement regarding the positive impact of the GPP role on patient outcomes. Similarly, most GPPs (98.1%, *n* = 152) acknowledged that the GPP role effectively mitigated work pressure within primary care. Furthermore, participants recognised the GPP’s contribution in reducing prescribing errors (96.8%, *n* = 150), saving NHS resources by freeing up GPs’ time (91.6%, *n* = 142), and minimising medicine waste (96.8%, *n* = 150) as indicated in Table 5.

**Table 5:** Views on the impact of GPPs in primary care

Statement	Agree/Strongly agree *N* (%)	Neither agree nor disagree *N* (%)	Disagree/Strongly disagree *N* (%)	Missing *N* (%)
The GPP role has a positive impact on patient outcomes.	151 (97.5)	1 (0.6)	1 (0.6)	2 (1.3)
GPPs help to alleviate work pressure within primary care.	152 (98.1)	0 (0)	1 (0.6)	2 (1.3)
GPPs help to reduce prescribing errors.	150 (96.8)	2 (1.3)	1 (0.6)	2 (1.3)
GPPs will save the NHS money by potentially freeing up GPs’ time.	142 (91.6)	9 (5.8)	2 (1.3)	2 (1.3)
Employing a GPP in a general practice will save the NHS money by reducing medicine waste.	150 (96.8)	2 (1.3)	1 (0.6)	2 (1.3)

GP: general practitioner, GPP: general practice pharmacist, NHS: National Health Service

The last question in the questionnaire solicited additional remarks from GPPs regarding their role and the overall impact of GPPs in general practice. A total of 61 replies were collected which were broadly grouped into categories. Most of the GPPs provided insights on the advantages that GPPs could offer to GPs and the broader field of general practice. For example, GPPs indicated that they had the potential to enhance the quality of patient care and improve patient experience. However, numerous participants noted challenges commonly faced by GPPs in the general practice. Some GPPs expressed uncertainty over the future development of the GPP role within the field of general practice and insufficient financial remuneration (pay). Table 6 presents results of analysis of the free text responses under several categories. Each text comment is accompanied with a representative quote.

**Table 6:** Free text comments from respondents on the main issues encountered by GPPs

Categorisation of free text comments	GPPs’ quotations
Limited patient-facing activities	*“… I free up significant amount of time for GPs, but this has not translated to more patients being seen…”* (GPP016)
Lack of training	*“I feel our role could have been developed better if the GPPs had specific training. Like the training given to the federation-practice nurses.”* (GPP108)
Different roles across practices	*“…there is too much variations (*sic*) between practices and what GPP are ‘allowed’ or encouraged to be involved in. Some GPs won’t consider IP practising, others will have GPP running everything-no consistency-difficult to know if you’re meeting your contracted obligations*.” (GPP064)
Promotion of GPP roles	“… *I personally think there should be more promotion at a national level with good examples of how GPP fit in + the benefits they bring.”* (GPP124)
More GPP posts	*“Additional GPPs are required to fulfil the workload pressures and facilitate upskilling and advanced roles of experienced GPPs.”* (GPP100)
Improvement in salaries	*“Pay scale needs addressed to reflect the work and responsibilities GPP undertake within general practice.”* (GPP140)
Mentor support	*“Valuable role but more support needed when transferring from community pharmacy/ other working role. Perhaps allocated a GPP mentor who has had years of experience- not a Lead but just a go to on similar level.”* (GPP148)
GPP’s employment	*“…GPPs should have been employed by HSCB/DOH, not federations.”* (GPP016)
GPP role development	*“Role of GPP needs to be extended further. Needs to be more established career progression. Extend GPP role to deliver more services within general practice. More training + learning should be made available.”* (GPP140)
Improve collaboration	*“I feel more could be done to improve collaboration within the MDT in primary care, as the number of GPs decline, we need to focus on better interprofessional collaboration across the widely extended primary care MDT.”* (GPP139)
GPP’s attitudes to their role	*“Whilst I agree that GPPs have a positive role on patient outcomes, this role is largely administrative. Most of my time is spent re-issuing acute prescriptions and processing discharge letters. Job satisfaction is poor and I am actively seeking work in another sector.”* (GPP081)
Public awareness of GPP	*“General public/patients still very unaware of role and can be surprised if GPP phones/does their clinic appointment.”* (GPP047)
The impact of the role of GPP	*“The impact role of a GPP is dependent on GP buy-in and co-operation. My second practice have never embraced my role and as a result my role is extremely limited, it is really a waste of time are being* (sic) *there as the GP thinks, he does not need me and said this to my face!”* (GPP014)

GP: general practitioner, GPP: general practice pharmacist, IP: independent prescriber, DOH: Department of Health, HSCB: Health and Social Care Board, MDT: multi-disciplinary team

## Discussion

### Summary

This study illustrated that most GPP participants undertook a range of activities, demonstrated positive attitudes towards collaboration with GPs and had positive views about their role impact in primary care.

### Comparison with literature

Demographic data of GPPs in this study related to age and gender were comparable with the demographic data of other published studies conducted on the same topic in the UK or elsewhere [10, 18, 21, 22]. These data were also consistent with the publicly available data relating to GPPs in NI at the time of this study, as most participants were female and were younger than 40 years of age [16].

Most GPPs were qualified to practise as independent prescribers, with over half using the independent prescriber qualification. This is consistent with previous research in NI and other parts of the UK [10, 15, 18, 22]. The Pharmacy Workforce Review in NI found that over 600 pharmacists were independent prescribers in 2020, with the majority working in secondary care [16]. However, this figure may change as the role of GPPs becomes established in primary care and pharmacist prescribing becomes a key activity [16]. The GPP prescribing role in general practice would appear to be limited at present, with some participants reporting that they were not currently prescribing for patients despite being qualified as independent prescribers. In this situation, GPPs are unable to make any modifications related to prescribing and such modifications must be done by or under the supervision of the GP [29]. Published research has indicated that barriers to pharmacist prescribing include inadequate training on specific knowledge and skills, insufficient assistance from authorities and stakeholders, and a lack of funding or reimbursement [30]. Additionally, the results from this study indicated that there was no association between working arrangements of the GPP (number of practices in which the GPP was working, and number of sessions worked per week) and the prescribing status of the GPP (if the GPP had an independent prescribing qualification or not and if the GPP was currently using the qualification or not). There may be other reasons that are more important than working arrangements that affect whether GPPs prescribe, and such data may not have been collected in this study, e.g. attitudes of GPPs to non-medical prescribing [30].

Over half of the GPP participants worked in multiple general practices. This reflects GP responses (38.7%) in a study in NI where they indicated that occasionally GPPs were not available in the general practice when they were required [15]. This has also been reported by patients stating that the GPP may not be in the general practice when they needed them [31]. Additionally, prior research has found that the restricted amount of time that GPPs dedicate to their practice, primarily due to working part-time, can hinder their integration, availability, and impact in general practice [18, 32, 33]. This finding emphasises how crucial it is for the general practice to have a full-time GPP [15].

More than half of GPPs reported that they ‘always’ or ‘frequently’ had a consulting room available for them to use. This is positive as unavailability of designated workplaces has been noted as a barrier to GPP integration in general practice [22, 33]. Most GPPs in this study had previous experience of working in community pharmacy. Similar results of GPPs’ previous work experience were also highlighted in other research studies conducted in the UK [10, 22]. The Pharmacy Workforce Review indicated that over the period from 2009 to 2020 the need for more pharmacists’ posts had grown in NI in both primary and secondary care [16]. This occurred because of new roles being established in general practice and hospitals implementing seven-day working each week [16]. Many of these new roles have been filled by community pharmacists, which reflects the previous working experience of GPP participants in this study [16].

Almost all GPPs performed patient-level activities, such as medication reviews and reconciliation. These are two essential activities carried out by GPPs in England, Scotland, and NI, based on findings from the literature [15, 18, 22]. By offering these two activities, medication errors—such as therapeutic duplication, dosage issues, or drug interactions—and inappropriate prescribing can be reduced [34]. Most participants indicated that activities such as these had been determined through mutual agreement between the GPP and GP, which aligns with the findings of the earlier GP study conducted in NI [15].

This study found that over 80% of GPPs reported face-to-face contact as the most common method of communication with GPs. This finding was similarly documented in a prior study conducted in NI, when all GP participants engaged in face-to-face meetings with GPPs [15]. This contrasts with an Australian study where GPPs mainly contacted GPs via telephone [21]. Face-to-face contact facilitates direct communication between GPPs and GPs, which is essential for managing a multidisciplinary approach to healthcare in primary care contexts [35]. Common issues discussed during GPP-GP meetings included medicines interactions, side effects, contraindications, starting new medicines, patients with comorbidities, audit results, and workload within the practice; these had been noted in previous research suggesting that they are the most important issues [15]. The main form of contact between GPPs and community pharmacists was via the telephone, with issues such as medicine alternatives, prescription queries from the practice, cost-effective choices, nursing home queries, and stock of specific items. These findings support previous research that found similar issues were often discussed during such contacts [36].

Most GPPs demonstrated positive attitudes towards collaboration with GPs, agreeing or strongly agreeing with all the ATCI-P statements. Statistical analysis showed that compared to GPPs who worked in a single practice, those who worked across many practices were more likely to agree or strongly agree with most of the ATCI-P statements. These results may indicate that GPPs who work in several practices are more adept at fostering relationships across various practices [22]. Additionally, these results corroborate those of a cross-sectional questionnaire study conducted in NI, which found that GPs’ attitudes towards working with GPPs were generally positive [15]. This latter study also used the Attitudes Towards Collaboration Instrument for GPs (ATCI-GP) to measure GPs’ attitudes towards collaboration with GPPs [15]. A survey study which assessed collaboration and team effectiveness of GPPs based on four components [professional interactions, relationship initiation, exchange characteristics (trust and role clarity), and commitment to collaboration] showed that GPPs’ collaboration and team effectiveness scores from the survey were high overall [37]. The authors of this survey study proposed that long-term employment and long working hours in general practice may promote interprofessional collaboration and team effectiveness through gradual improvement of trust and working relationships between GPPs and other HCPs [37]. Based on the findings of this present study on the working arrangements of GPPs and ATCI-P responses (i.e. most GPPs work in more than one general practice), it seems that GPPs working in several general practices may enhance their relationships with other HCPs in those practices, including GPs [22]. Nevertheless, it is still essential to have a dedicated full-time GPP in every general practice to promote GPP integration, availability, interprofessional collaboration with HCPs and impact within the general practice [15, 18, 37].

The study found that telephone contact was the most common and preferred method of communication between GPPs and patients in general practice. This is consistent with previous research in NI, where GPs’ responses indicated that using the telephone was the most common and preferred method of contact with patients [15]. Furthermore, based on the GPPs’ response in the present study, just over one-third of GPPs thought that patients were aware of their role, which may explain why patients sometimes hesitate to accept and schedule appointments with the GPPs [15, 22]. However, most participants agreed that the professional activities of GPPs were valued by patients, which is consistent with literature reporting patients’ high levels of satisfaction with the quality of care received from GPPs [38–40].

The majority of GPPs agreed that the GPP role had a positive impact on patient outcomes and alleviated work pressure within primary care. This could be as a result of the GPP’s ability to prescribe medicines for patients as shown in the results of this study. The advantages of allowing GPPs to prescribe have been noted before [41, 42], including better use of GPP skills and expertise and improving patient care [41, 42]. Additionally, work pressure in primary care has been alleviated by GPPs and nurses managing conditions that need long-term care which allows the GPs to focus on conditions of acute care [43].

Free-text responses emphasised the potential benefits that GPPs could have for GPs and general practice. For instance, GPPs reported that they could improve patient care and experience, optimise prescribing, decrease adverse effects, and free up the GP’s time. Analysis of these comments also indicated the need for more GPP patient-facing activities, training tailored to GPPs, promotion of the role to patients, better salaries, mentor support, more GPP sessions, more full-time positions, and changes to GPP employment procedures. Prior research also identified a number of these needs, highlighting how critical it is to address these issues [15].

### Strengths and limitations

The current study had a response rate of 48.5%, which was greater than that of some previous studies that were published on the same topic [10, 21]. Utilising a self-administered questionnaire offered a cost-efficient method for gathering data from a specific population, while ensuring enhanced anonymity due to the absence of direct interpersonal engagement with participants [44, 45]. Furthermore, by giving participants the option to reply independently without being influenced by the interviewer’s presence, as in the case of interview-administered questionnaires, the administration method (postal questionnaires) and completion method (self-completion) may have reduced response bias [46]. No reliability or validity assessment was performed on the questionnaire [47]. Certain findings (e.g. features of collaboration between GPPs and GPs) may not apply to other UK regions, as the study sample consisted only of NI-based GPPs, thereby limiting generalisability of the findings. Moreover, extrapolating the study’s conclusions to the GPPs in NI who declined to participate might be challenging. Nonetheless, most of the results of this study reflected those of other national and international studies.

## Conclusion

This study has revealed that GPP views on collaboration with GPs were generally favourable, there was variation in the role of the GPP and how activities were agreed upon, GPPs were positive about their impact on primary care, and recognised that communication with patients needed to improve, along with enhancing patient awareness of the GPP. As the role of GPPs becomes more established in general practice, the results of this study might offer practical information for policies and service commissioners.

### Electronic supplementary material

Below is the link to the electronic supplementary material.

Supplementary Material 1. Additional file 1. CROSS checklist

Supplementary Material 2. Additional file 2. The study questionnaire

Supplementary Material 3. Additional file 3. Communication of GPP

Supplementary Material 4. Additional file 4. Reasons of GPP communication

Supplementary Material 5. Additional file 5. Associations results

## Data Availability

The corresponding author will make the data underpinning this article available upon reasonable request.

## Abbreviations

ATCI-P: Attitudes Towards Collaboration Instrument for pharmacists
EMIS®: Egton Medical Information Systems
GPs: General practitioners
GPPs: General practice pharmacists
HCPs: healthcare professionals
NHS: National Health Service
NI: Northern Ireland
PCNs: Primary Care Networks
UK: United Kingdom
