# Supplementary material for: An exploration of Northern Ireland general practice pharmacists’ views on their role in general practice: a cross-sectional survey
Source: BMC Prim Care. 2024 Jun 6;25:201. doi: 10.1186/s12875-024-02457-7 (PMC11157875; doi:10.1186/s12875-024-02457-7)
Supplement: Supplementary file 2 — Supplementary Material 2. Additional file 2. The study questionnaire [file 12875_2024_2457_MOESM2_ESM.docx]

**An exploration of Northern Ireland general practice pharmacists’ (GPPs) views on their role in general practice: a cross-sectional questionnaire study**

| Section A | Demographic information |
| --- | --- |
| Section B | Activities of general practice pharmacists |
| Section C | General practice pharmacists’ communication within primary care |
| Section D | Attitudes towards collaboration with general practitioners |
| Section E | General practice pharmacists’ views on communication with patients and patients’ awareness of their role |
| Section F | Views on the impact of general practice pharmacists in primary care |

**How to complete this questionnaire**

- This questionnaire should take approximately 15 minutes to complete.
- This questionnaire should be completed by a GPP who spends most time in the general practice.
- If you work in more than one general practice, you should only complete the questionnaire once and the responses should reflect your experience of the general practice at which you spend the most time.
- If you spend your time equally between different general practices, you should only complete the questionnaire once and the responses should reflect your experience at one of these general practices.
- You should avoid reporting any identifiable information in your responses to questionnaire questions of general practitioners, community pharmacists, patients and other practice staff.
- All the information gathered will be anonymous and cannot be linked to you as an individual. There are no right or wrong answers and all answers are useful. We are interested in your personal views, not what you think we want to hear.

| **SECTION A: DEMOGRAPHIC INFORMATION** |
| --- |

*This section of the questionnaire is concerned with gathering some details about you and your work. Please read the statements below and confirm by ticking one or more box(es) that apply to you.*

1. **What is your gender? (Please select ONE option)**

| Female | Male | Prefer not to say | *Other |
| --- | --- | --- | --- |
| 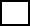 | 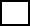 | 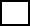 | 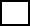 |

*Please specify below:

1. **What is your age (in years)? (Please select ONE option)**

| $<$30 | 30–39 | 40–49 | 50–59 | $\geq$60 |
| --- | --- | --- | --- | --- |
| 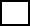 | 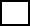 | 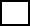 | 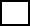 | 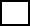 |

1. **Have you completed a postgraduate qualification? e.g. Diploma, MSc**

**(Please select ONE option)**

| Yes* | No |
| --- | --- |
| 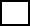 | 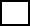 |

*** If you have completed a postgraduate qualification, please specify below:**

1. **Do you have an Independent Prescriber qualification? (Please select ONE option)**

| Yes  (Go to Q5) | No  (Go to Q6) |
| --- | --- |
| 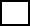 | 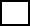 |

1. **Are you currently using the Independent Prescriber qualification? (Please select ONE option)**

| Yes | No |
| --- | --- |
| 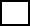 | 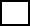 |

1. **In how many general practices do you work? (Please select ONE option)**

| 1 | 2 | 3 | $>$3 |
| --- | --- | --- | --- |
| 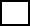 | 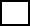 | 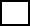 | 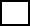 |

**7. How many sessions per week do you work at the general practice in which you spend most of your time? Please specify below:**

**8. How long have you been working as a GPP (in years)? (Please select ONE option)**

| $<$1 | 1-4 | 5-9 | $\geq$10 |
| --- | --- | --- | --- |
| 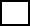 | 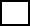 | 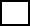 | 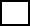 |

1. **How long have you been working at the general practice in which you spend most of your time (in years)? (Please select ONE option)**

| $<$1 | 1-4 | 5-9 | $\geq$10 |
| --- | --- | --- | --- |
| 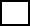 | 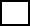 | 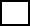 | 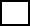 |

1. **How often is a consulting room available for you to use at the general practice in which you spend most of your time? (Please select ONE option)**

| Always | Very often | Sometimes | Rarely | Never |
| --- | --- | --- | --- | --- |
| 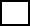 | 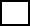 | 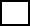 | 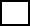 | 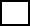 |

1. **In which Trust area of Northern Ireland is the general practice in which you spend most of your time located? (Please select ONE option)**

| Belfast | Northern | South-Eastern | Southern | Western |
| --- | --- | --- | --- | --- |
| 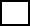 | 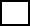 | 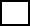 | 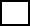 | 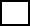 |

1. **How would you describe the location of the general practice in which you spend most of your time? (Please select ONE option)**

| Rural | Suburban | Urban |
| --- | --- | --- |
| 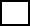 | 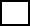 | 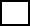 |

1. **In what other sectors of pharmacy have you worked? Please tick all that apply:**

| Community pharmacy | Hospital pharmacy | Academia | Pharmaceutical industry | *Other |
| --- | --- | --- | --- | --- |
| 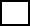 | 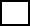 | 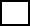 | 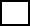 | 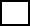 |

*Please specify below:

| **SECTION B: ACTIVITIES OF GENERAL PRACTICE PHARMACISTS** |
| --- |

*This section will focus on activities you provide at the general practice. Please read the statements below and confirm by ticking one or more box(es) that apply to you.*

1. **Which activities do you provide within the practice in which you spend most of your time? Please tick all that apply:**

| Medication reviews. | 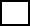 |
| --- | --- |
| Medication reconciliation. | 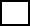 |
| Triaging and managing minor ailments. | 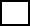 |
| Counselling patients to help them manage their medications. | 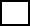 |
| Educating patients on how to take their medicines. | 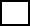 |
| Educational group sessions to patients. | 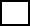 |
| Counselling patients in relation to lifestyle interventions. | 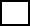 |
| Addressing medicines adherence with patients. | 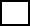 |
| Patient medication queries. | 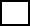 |
| Acute prescribing. | 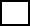 |
| Reauthorising repeat prescribing. | 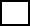 |
| Running clinics with patients (e.g. asthma, blood pressure, vaccination). | 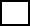 |
| Signposting patients to appropriate services and other healthcare professionals (e.g. community pharmacists). | 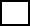 |
| Managing other issues that involve medication such as adverse drug reactions and drug-drug interactions. | 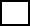 |
| Educational group sessions to health care providers. | 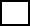 |
| Answering medicines information enquiries from health care providers. | 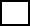 |
| Student training. | 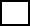 |
| Conducting audits as part of the multidisciplinary team. | 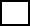 |
| Administrative duties such as dealing with outpatient clinical letters and hospital discharge letters. | 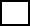 |
| Developing guidelines and/or practice formulary. | 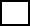 |
| Research. | 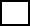 |
| Outreach involvement (e.g Drug and Therapeutics Committee). | 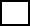 |

Please list below any other activities undertaken by you:

|  |
| --- |
|  |
|  |

1. **How were your activities decided upon in the general practice in which you spend most of your time? Please tick all that apply:**

| Through mutual agreement between you and the GP. | 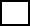 |
| --- | --- |
| Determined by your current skills. | 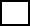 |
| Determined by your level of confidence. | 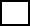 |
| Determined by your previous experience. | 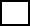 |
| Determined by GP Federation. | 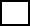 |
| Other, please specify below: | 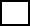 |
|  |  |

|  |
| --- |

| **SECTION C: GENERAL PRACTICE PHARMACISTS’ COMMUNICATION WITHIN PRIMARY CARE** |
| --- |

*The following questions focus on your communication with general practitioners (GPs), community pharmacists and other staff within the general practice. Please read the statements below and confirm by ticking one or more box(es) that apply to you.*

***Communication with general practitioners (GPs)***

1. **What is/are the most common method(s) of communication between yourself and GPs in the general practice in which you spend most of your time? Please tick all that apply:**

| Email | Face-to-face | Instant messaging | Telephone | Written | Other* |
| --- | --- | --- | --- | --- | --- |
| 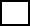 | 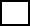 | 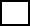 | 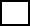 | 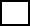 | 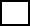 |

*Please specify below:

1. **What is/are the most preferred method(s) of communication between yourself and the GPs in the general practice in which you spend most of your time? Please tick all that apply:**

| Email | Face-to-face | Instant messaging | Telephone | Written | Other* |
| --- | --- | --- | --- | --- | --- |
| 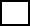 | 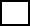 | 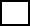 | 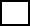 | 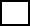 | 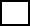 |

*Please specify below:

1. **If you meet face-to-face, on average, how often do you meet with the GPs in the general practice in which you spend most of your time? (Please select ONE option)**

| Daily | 2-3 times/ week | Once a week | Once a fortnight | Once a month | Other* |
| --- | --- | --- | --- | --- | --- |
| 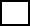 | 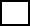 | 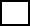 | 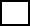 | 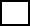 | 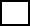 |

*Please specify below:

1. **What are the most common reasons for you to communicate with the GPs in the general practice in which you spend most of your time? Please list the reasons below:**

|  |
| --- |
|  |

1. **What are the most common reasons for the GPs in the general practice in which you spend most of your time to communicate with you? Please list the reasons below:**

|  |
| --- |
|  |

***Communication with community pharmacists***

1. **What is/are the most common method(s) of communication between yourself and the community pharmacists with whom you have most contact? Please tick all that apply:**

| Email | Face-to-face | Instant messaging | Telephone | Written | Other* |
| --- | --- | --- | --- | --- | --- |
| 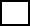 | 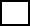 | 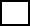 | 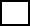 | 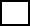 | 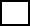 |

*Please specify below:

1. **What is/are the most preferred method(s) of communication between yourself and the community pharmacists with whom you have most contact? Please tick all that apply:**

| Email | Face-to-face | Instant messaging | Telephone | Written | Other* |
| --- | --- | --- | --- | --- | --- |
| 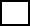 | 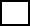 | 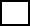 |  |  |  |

*Please specify below:

1. **On average, how often do you have face-to-face contact with community pharmacists with whom you have most contact? Please skip this question and go to Q24 if you do not have face-to-face contact with community pharmacists:**

| Daily | 2-3 times/ week | Once a week | Once a fortnight | Once a month | Other* |
| --- | --- | --- | --- | --- | --- |
|  |  |  |  |  |  |

*Please specify below:

1. **What are the most common reasons for you to communicate with the community pharmacists with whom you have most contact? Please list the reasons below:**

|  |
| --- |
|  |

1. **What are the most common reasons for the community pharmacists with whom you have most contact to communicate with you? Please list the reasons below:**

|  |
| --- |
|  |

***Communication with other practice staff***

1. **What other health and social care professionals communicate with you in the general practice in which you spend most time? Please circle ‘Yes’ or ‘No’. If you circle ‘Yes’, please provide the number of other health and social care professionals in the space below:**

| **Health and social care professionals** | **Circle yes or no** | **Number of health and social care professionals** |
| --- | --- | --- |
| Pharmacy technician | Yes / No |  |
| Practice nurse | Yes / No |  |
| Reception staff | Yes / No |  |
| Practice managers | Yes / No |  |
| Other, please specify below: | Yes / No |  |
|  |  |  |

| **SECTION D: ATTITUDES TOWARDS COLLABORATION WITH GENERAL PRACTITIONERS** |
| --- |

*Please indicate the extent to which you agree or disagree with the following statements by placing a tick (*$\surd$*) in the appropriate box:*

|  | Strongly disagree | Disagree | Neither agree nor disagree | Agree | Strongly agree |
| --- | --- | --- | --- | --- | --- |
| 1. **The professional communication between myself and the GP is open and honest.** |  |  |  |  |  |
| 1. **The GP is open to working together with me on patients’ medication management.** |  |  |  |  |  |
| 1. **The GP has time to discuss with me matters relating to patients’ medication regimens.** |  |  |  |  |  |
| 1. **I meet the professional expectations of the GP.** |  |  |  |  |  |
| 1. **The GP trusts my professional decisions.** |  |  |  |  |  |
| 1. **Discussions with the GP help me provide better patient care.** |  |  |  |  |  |
| 1. **The GP and I have mutual respect for one another on a professional level.** |  |  |  |  |  |
| 1. **The GP and I share common goals and objectives when caring for the patient.** |  |  |  |  |  |
| 1. **My role and the GP’s role in patient care are clear.** |  |  |  |  |  |

|  | Strongly disagree | Disagree | Neither agree nor disagree | Agree | Strongly agree |
| --- | --- | --- | --- | --- | --- |
| 1. **The GP has confidence in my expertise.** |  |  |  |  |  |
| 1. **The GP believes that I have a role in assuring medication safety (for example, to identify drug interactions, adverse reactions, contraindications etc).** |  |  |  |  |  |
| 1. **The GP believes that I have a role in assuring medication effectiveness (for example, to ensure the patient receives the optimal drug at the optimal dose etc).** |  |  |  |  |  |
| 1. **My working together with the GP benefits the patient.** |  |  |  |  |  |

| **SECTION E: GENERAL PRACTICE PHARMACISTS’ VIEWS ON COMMUNICATION WITH PATIENTS AND PATIENTS’ AWARENESS OF THEIR ROLE** |
| --- |

*Questions in this section will focus on your communication with patients and your views on patients’ awareness of the GPP role. Please read the statements below and confirm by ticking one or more box(es) that apply to you.*

1. **What is/are the most common method(s) of communication between yourself and patients in the general practice in which you spend most of your time? Please tick all that apply:**

| Email | Face-to-face | Instant messaging | Telephone | Written | Other* |
| --- | --- | --- | --- | --- | --- |
|  |  |  |  |  |  |

*Please specify below:

1. **What is/are the most preferred method(s) of communication between yourself and patients in the general practice in which you spend most of your time? Please tick all that apply:**

| Email | Face-to-face | Instant messaging | Telephone | Written | Other* |
| --- | --- | --- | --- | --- | --- |
|  |  |  |  |  |  |

*Please specify below:

1. **On average, how often do you have face-to-face contact with patients at the general practice in which you spend most of your time? Please skip this question and go to Q44 if you do not have face-to-face contact with patients:**

| Daily | 2-3 times/ week | Once a week | Once a fortnight | Once a month | Other* |
| --- | --- | --- | --- | --- | --- |
|  |  |  |  |  |  |

*Please specify below:

|  |
| --- |

1. **If you meet face-to-face with patients, what are the main issues that you usually discuss? Please list the issues below:**

|  |
| --- |
|  |

1. **Please indicate the extent to which you agree or disagree with the following statements by placing a tick (**$\boldsymbol{\surd}$**) in the appropriate box:**

|  | Strongly disagree | Disagree | Neither agree nor disagree | Agree | Strongly agree |
| --- | --- | --- | --- | --- | --- |
| Patients are aware of the role I provide. |  |  |  |  |  |
| Patients are aware of the difference between the GPP role and the community pharmacist role. |  |  |  |  |  |
| Patients trust my ability to provide high-quality care. |  |  |  |  |  |
| My professional activities are valued by patients. |  |  |  |  |  |

| **SECTION F: VIEWS ON THE IMPACT OF GENERAL PRACTICE PHARMACISTS IN PRIMARY CARE** |
| --- |

*Please indicate the extent to which you agree or disagree with the following statements about general practice pharmacists (GPPs) by placing a tick (*$\surd$*) in the appropriate box:*

|  | Strongly disagree | Disagree | Neither agree nor disagree | Agree | Strongly agree |
| --- | --- | --- | --- | --- | --- |
| 1. The GPP role has a positive impact on patient outcomes. |  |  |  |  |  |
| 1. GPPs help to alleviate work pressure within primary care. |  |  |  |  |  |
| 1. GPPs help to reduce prescribing errors. |  |  |  |  |  |
| 1. GPPs will save the NHS money by potentially freeing up GPs’ time. |  |  |  |  |  |
| 1. Employing a GPP in a general practice will save the NHS money by reducing medicine waste. |  |  |  |  |  |

1. **Do you have any further comments you wish to make about your role as a GPP and your impact in general practice?**

|  |
| --- |
|  |
|  |
|  |

***Thank you very much for your time and interest in completing the questionnaire***
