# Supplementary material for: An exploration of Northern Ireland general practice pharmacists’ views on their role in general practice: a cross-sectional survey
Source: BMC Prim Care. 2024 Jun 6;25:201. doi: 10.1186/s12875-024-02457-7 (PMC11157875; doi:10.1186/s12875-024-02457-7)
Supplement: Supplementary file 3 — Supplementary Material 3. Additional file 3. Communication of GPP [file 12875_2024_2457_MOESM3_ESM.docx]

GPP-GP communication as reported by GPPs participants


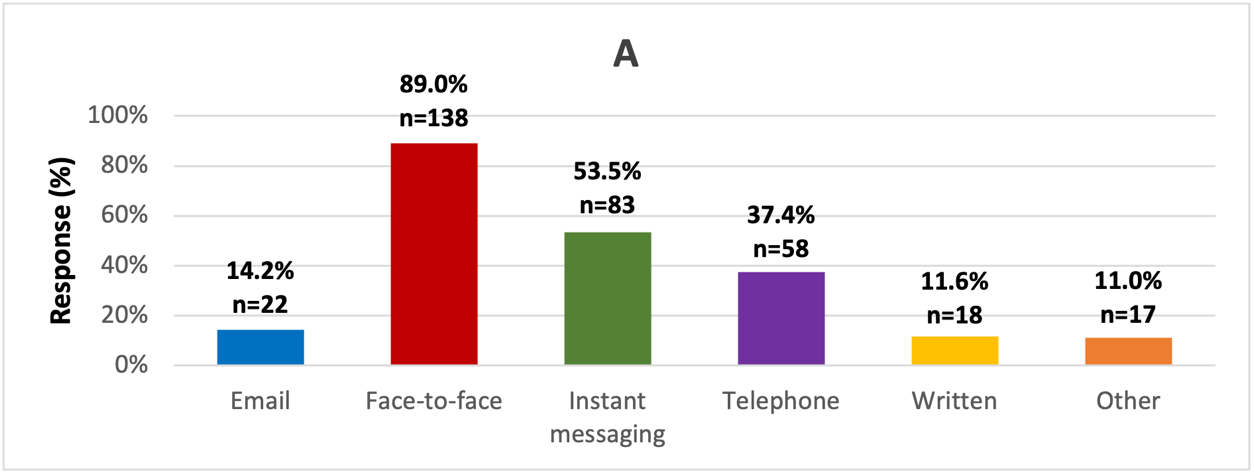

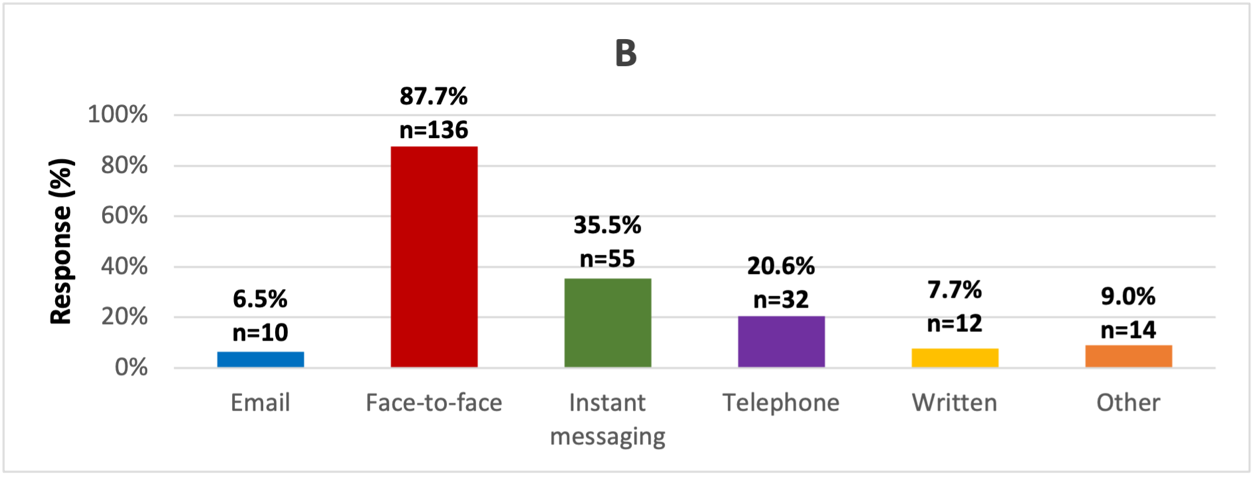


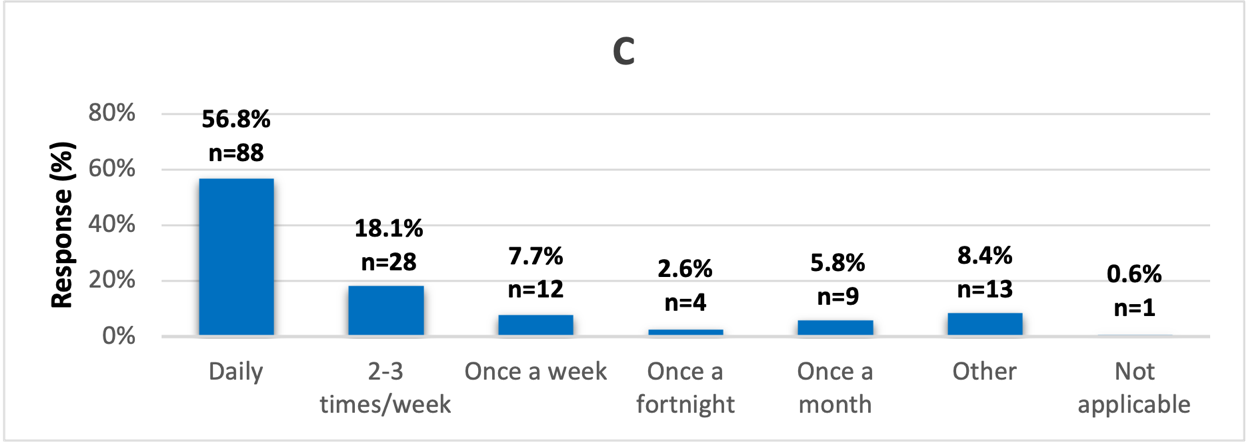


**Figure A. The most common method(s) of communication between GPPs and GPs**

**Figure B. The most preferred method(s) of communication between GPPs and GPs**

**Figure C. Frequency of face-to-face meetings between GPPs and GPs**

GPP-Community pharmacist communication as reported by GPPs participants


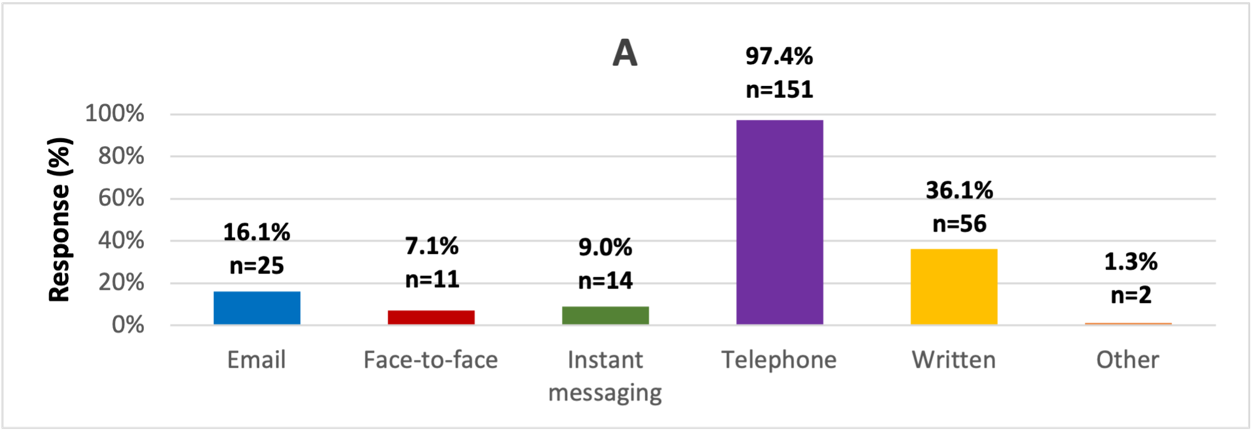

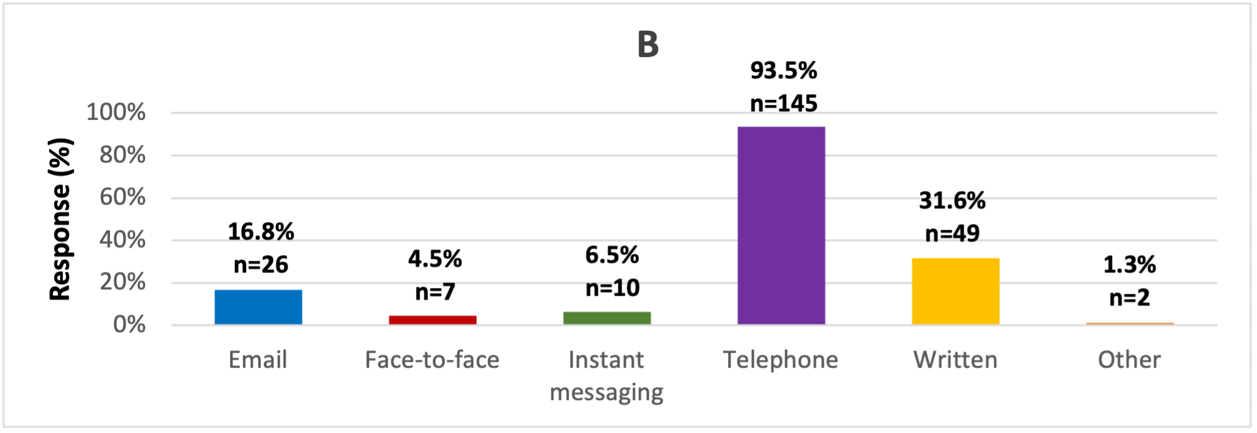


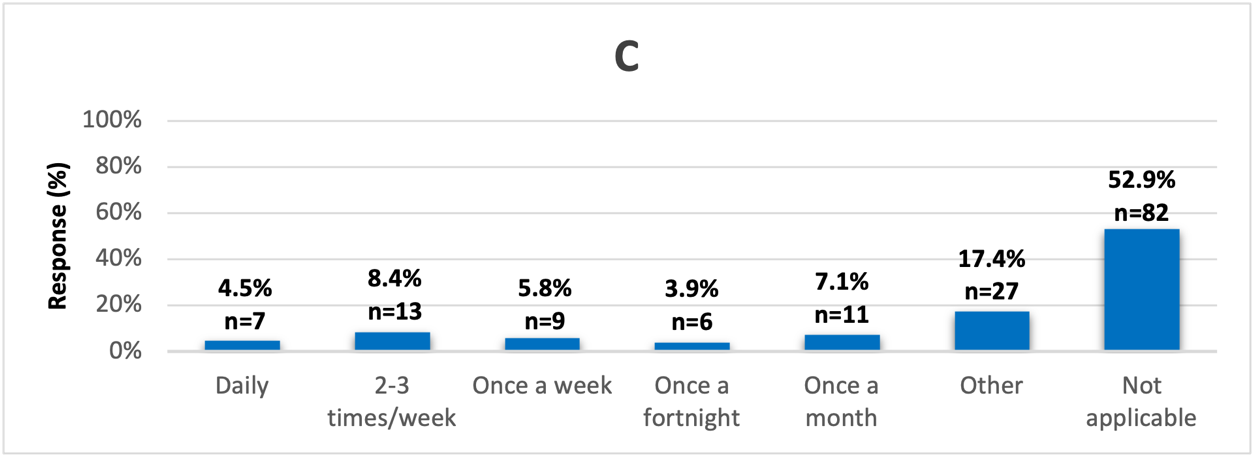


**Figure A. The most common method(s) of communication between GPPs and community pharmacists**

**Figure B. The most preferred method(s) of communication between GPPs and community pharmacists**

**Figure C. Frequency of face-to-face meetings between GPPs and community pharmacists**

GPP-Health and social care professionals communication as reported by GPPs participants

**
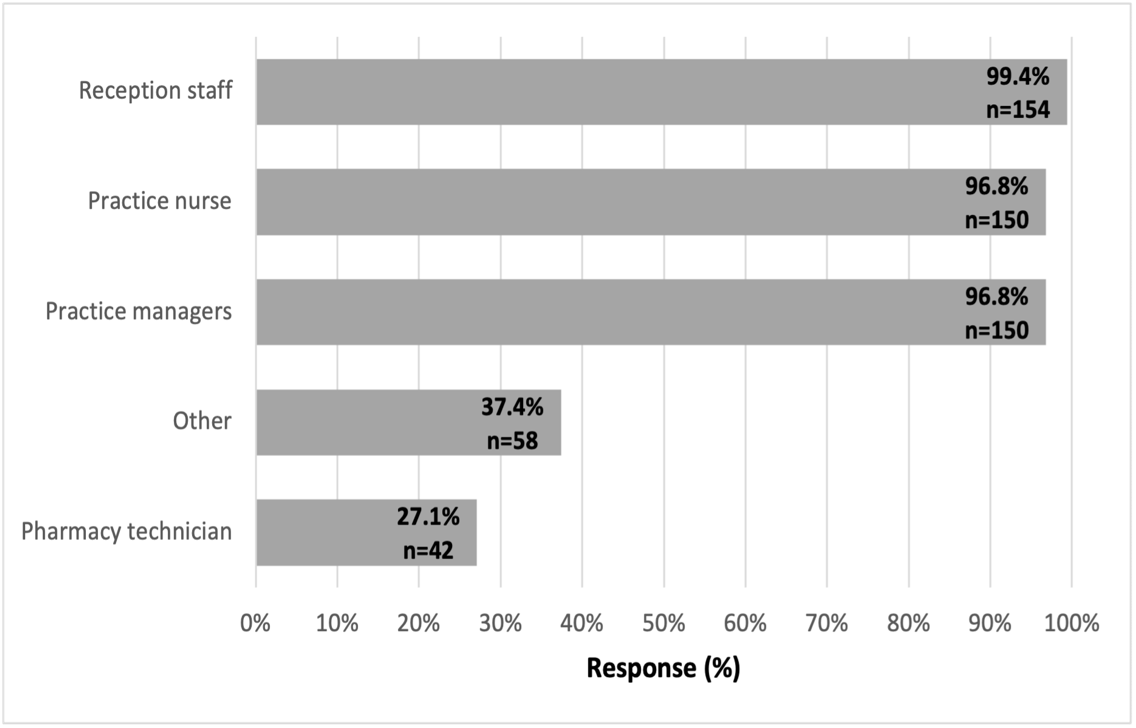
**

**Figure A. GPP communication with other health and social care professionals**

GPP-Patient communication as reported by GPPs participants


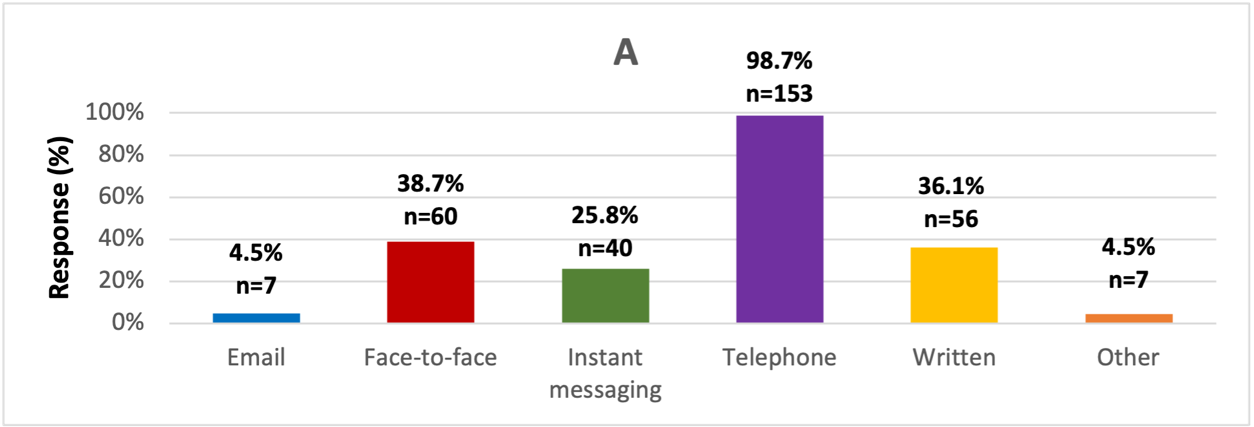


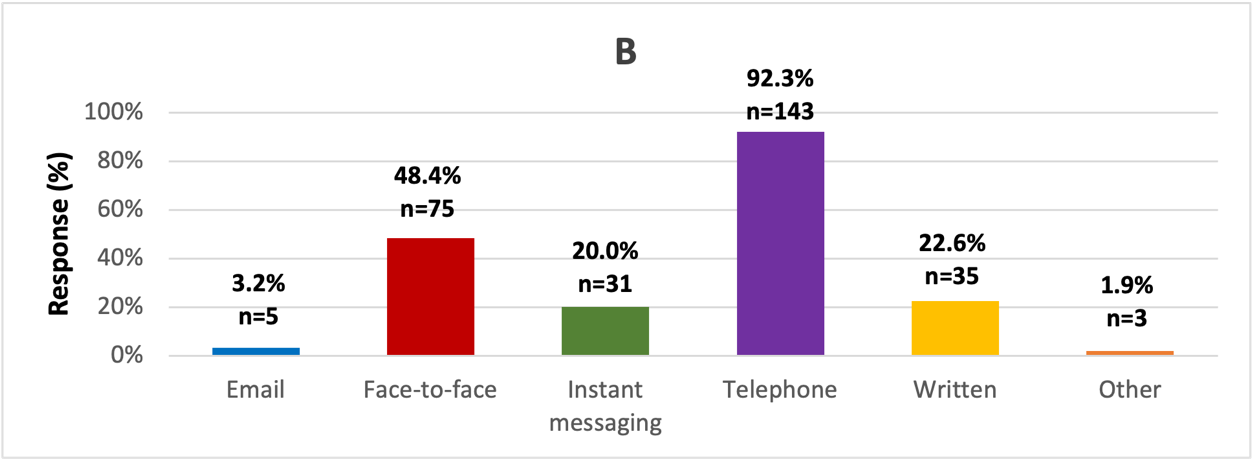


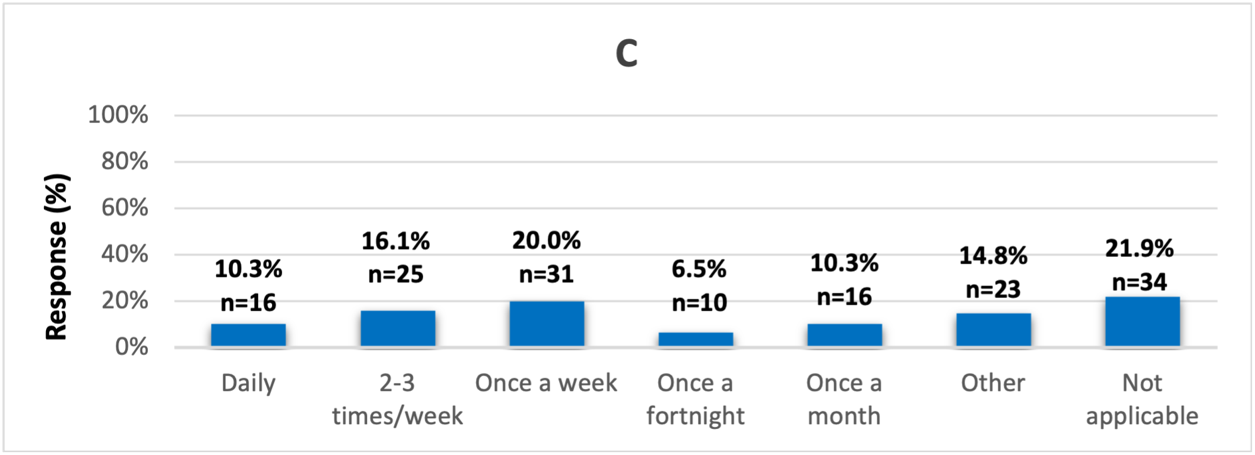


**Figure A. The most common method(s) of communication between GPPs and patients**

**Figure B. The most preferred method(s) of communication between GPPs and patients**

**Figure C. Frequency of face-to-face meetings between GPPs and patients**
