# Supplementary material for: An exploration of Northern Ireland general practice pharmacists’ views on their role in general practice: a cross-sectional survey
Source: BMC Prim Care. 2024 Jun 6;25:201. doi: 10.1186/s12875-024-02457-7 (PMC11157875; doi:10.1186/s12875-024-02457-7)
Supplement: Supplementary file 4 — Supplementary Material 4. Additional file 4. Reasons of GPP communication [file 12875_2024_2457_MOESM4_ESM.docx]

The most common reasons for GPP-GP communication according to GPPs’ responses

| **Common reasons** | **Examples of reasons for GPP to communicate with GP** | **Examples of reasons for GP to communicate with GPP** |
| --- | --- | --- |
| Medication issues | To discuss interacting medications | To request medication advice or medication review |
| Prescribing issues | To check appropriateness of prescribing certain unlicenced medications | To consider stopping or starting new medications |
| Patient issues | To discuss patient’s mental health deterioration | To discuss patients with comorbidities |
| Transitions between care sectors | To discuss issues with correspondence from secondary care | To ask to follow-up queries with the hospital |
| Audit and COMPASS report | To discuss audit results | To ask to assist with COMPASS reports |
| Others | To discuss practice policy and guidelines for prescribing medications | To check latest Health and Social Care Board (HSCB) guidance |

COMPASS: A prescribing information system developed to provide GPs with feedback on their prescribing

The most common reasons for GPP-community pharmacist communication according to GPPs’ responses

| **Common reasons** | **Examples of reasons for GPP to communicate with community pharmacist** | **Examples of reasons for community pharmacist to communicate with GPP** |
| --- | --- | --- |
| Medication issues | To advise on interactions highlighted by community pharmacist | To query dose of specific medication |
| Dispensing issues | To discuss changes to weekly dispensed medication | To discuss changes to multiple dispensing |
| Prescription issues | To discuss acute urgent prescriptions | To query prescriptions from practice |
| Nursing home issues | To discuss nursing home patients’ queries | To discuss nursing home patients’ queries |
| Transition between care issues | To discuss changes in medications from hospital discharge letters | To discuss ECR letters from hospitals |
| Others | To check stock of specific items in a particular branch | To discuss cost-effective choices |

ECR: Electronic care record
