# Supplementary material for: An exploration of Northern Ireland general practice pharmacists’ views on their role in general practice: a cross-sectional survey
Source: BMC Prim Care. 2024 Jun 6;25:201. doi: 10.1186/s12875-024-02457-7 (PMC11157875; doi:10.1186/s12875-024-02457-7)
Supplement: Supplementary file 5 — Supplementary Material 5. Additional file 5. Associations results [file 12875_2024_2457_MOESM5_ESM.docx]

**Associations between number of general practices at which GPPs worked and ATCI-P statements**

| **Statements** | **p-value** |
| --- | --- |
| 1. The professional communication between myself and the GP is open and honest. | p<0.05 |
| 1. The GP is open to working together with me on patients’ medication management. | p<0.05 |
| 1. The GP has time to discuss with me matters relating to patients’ medication regimens. | p<0.05 |
| 1. I meet the professional expectations of the GP. | p<0.05 |
| 1. The GP trusts my professional decisions. | p<0.05 |
| 1. Discussions with the GP help me provide better patient care. | p>0.05 |
| 1. The GP and I have mutual respect for one another on a professional level. | p<0.05 |
| 1. The GP and I share common goals and objectives when caring for the patient. | p<0.05 |
| 1. My role and the GP’s role in patient care are clear. | p<0.05 |
| 1. The GP has confidence in my expertise. | p<0.05 |
| 1. The GP believes that I have a role in assuring medication safety. | p<0.05 |
| 1. The GP believes that I have a role in assuring medication effectiveness. | p<0.05 |
| 1. My working together with the GP benefits the patient. | p>0.05 |

GP: general practitioner
